# Evidence of Macroalgal Colonization on Newly Ice-Free Areas following Glacial Retreat in Potter Cove (South Shetland Islands), Antarctica

**DOI:** 10.1371/journal.pone.0058223

**Published:** 2013-03-04

**Authors:** María Liliana Quartino, Dolores Deregibus, Gabriela Laura Campana, Gustavo Edgar Juan Latorre, Fernando Roberto Momo

**Affiliations:** 1 Departamento de Biología Costera, Instituto Antártico Argentino, Buenos Aires, Argentina; 2 Museo Argentino de Ciencias Naturales “B. Rivadavia”, Buenos Aires, Argentina; 3 Departamento de Ciencias Básicas, Universidad Nacional de Luján, Luján, Argentina; 4 Facultad de Ciencias Naturales y Museo, Universidad Nacional de La Plata, La Plata, Argentina; 5 Universidad Nacional de General Sarmiento, Instituto de Ciencias, Los Polvorines, Argentina; The Pennsylvania State University, United States of America

## Abstract

Climate warming has been related to glacial retreat along the Western Antarctic Peninsula. Over the last years, a visible melting of Fourcade Glacier (Potter Cove, South Shetland Islands) has exposed newly ice-free hard bottom areas available for benthic colonization. However, ice melting produces a reduction of light penetration due to an increase of sediment input and higher ice impact. Seventeen years ago, the coastal sites close to the glacier cliffs were devoid of macroalgae. Are the newly ice-free areas suitable for macroalgal colonization? To tackle this question, underwater video transects were performed at six newly ice-free areas with different degree of glacial influence. Macroalgae were found in all sites, even in close proximity to the retreating glacier. We can show that: 1. The complexity of the macroalgal community is positively correlated to the elapsed time from the ice retreat, 2. Algae development depends on the optical conditions and the sediment input in the water column; some species are limited by light availability, 3. Macroalgal colonization is negatively affected by the ice disturbance, 4. The colonization is determined by the size and type of substrate and by the slope of the bottom. As macroalgae are probably one of the main energy sources for the benthos, an expansion of the macroalgal distribution can be expected to affect the matter and energy fluxes in Potter Cove ecosystem.

## Introduction

**Figure 1:**
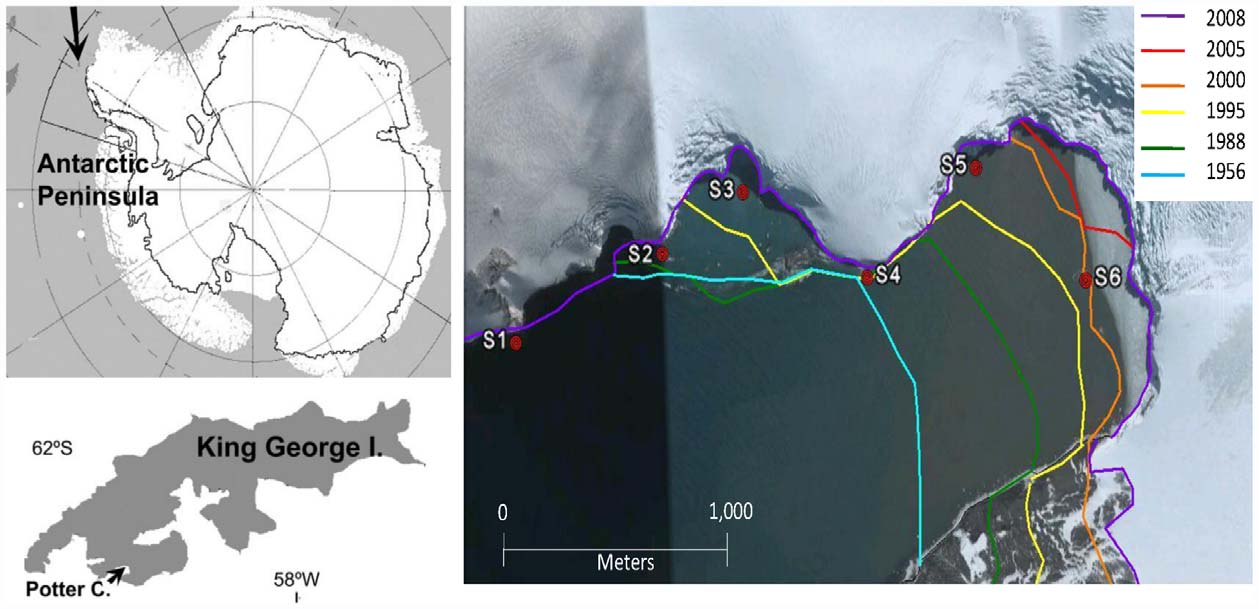
Maps of the location of 25 de Mayo/King George Island (KGI) on the Western Antartic Peninsula (arrow), Potter Cove on KGI (arrow) and satellite image of inner Potter Cove (Google Earth, 2011). Lines mark the retreat of Glacier Fourcade since 1956 [5]. Dots mark the six newly ice free areas sampling points (S1: site 1, S2: site 2, S3: site 3, S4: site 4, S5: site 5 and S6: site 6).

Antarctica is one of the regions most seriously affected by climate change; particularly, the Western Antarctic Peninsula is exhibiting a rapid regional warming [1]. Therefore, the glacial systems have shown a direct response to the higher temperatures with a marked melting and consequent retreat [2]. Continental ice melting can contribute to reduce light availability due to increasing sediment input [3]. Furthermore, an increase of the seafloor disturbance by scouring of ice blocks is expected in coastal areas [4]. One particular example of this situation was observed at Potter Cove (25 de Mayo/King George Island), where over the last years a visible melting of Fourcade glacier, the only glacier surrounding the Cove, has exposed several newly ice-free areas [5] (Figure 1).

The term “newly ice-free areas” refers to those places available for colonization and biological succession, due to glacier retreat [5]. The areas with hard substrate (e.g. rocks and boulders) are particularly suitable for macroalgal colonization. Macroalgal communities play a key role in the Antarctic coastal ecosystem. They are important primary producers, constituting food supply for benthic organisms, and represent a significant contribution to the particulate and dissolved organic matter for the coastal food web [6], [7]. Furthermore, macroalgae provide habitat and structural refuges [8]. Studies in Potter Cove revealed a complex macroalgal community with high biomass production, restricted to the outer, hard bottom coastal areas [9], [10]. At present, macroalgae are expected to be favored by the presence of new hard substrate for colonization. However, the impact of sediment in the water column could alter the light availability and modify their physiological responses.

According to the recent observations of the glacier retreat in Potter Cove, these questions arise: Is macroalgal distribution expanding into the newly ice-free areas? Which species are colonizing these areas? Is the structure of the communities related to the prevailing physical conditions in these areas?

To tackle these questions, underwater video transects were performed at six newly ice-free areas to assess the spatial macroalgal distribution and to compare the present situation with a study performed seventeen years ago [9].

## Methods

### Study Site

The study was carried out at Potter Cove (62° 14′ S, 58° 38′ W), from March to August 2008. The Cove is divided into an outer and an inner part. The bottom of the outer Cove consists of hard substrate whereas the inner part is a soft one. The southern shore of the inner Cove is a sandy beach, where creeks discharge. Glacier cliffs reach the Cove in the north and east [9].

Previous studies have shown that the rocky shores of the outer side of Potter Cove are colonized by a high biomass of macroalgae [9], [10], whereas the inner Cove has one of the largest concentrations of benthic filter feeders found in Antarctic coastal areas [11]. A clock-wise circulation characterizes the waters in the cove [3], with particle-free waters entering from its south western part. Creeks and glacial melting add particles to the inner cove, in relation with increasing air temperature. The presence of particles greatly limits light penetration in the water column which combined with wind-induced mixing explains the usually low phytoplankton production [12]. Since 1997 newly ice-free areas were detected gradually at Potter Cove. Six different sites (Sites 1 to 6) among these areas were selected according to their different degree of glacial influence and age of free ice release (Figure 1) [5]. Specifically, Site 6 is a small rocky island.

### Quantum-irradiance Measurements

Underwater photosynthetically active radiation (PAR, 400–700 nm) was considered a proxy for glacial sediment input. Photosynthetically active radiation (PAR) was measured in the six different selected newly ice-free areas (Sites 1 to 6) using a Licor LI 1400 datalogger. Instantaneous PAR data (µmol m^−2^ s^−1^) was obtained at 0, 5, 10, and 20 m depth in summer 2009–2010 (December-March), data was measured weekly around noon. Kd, the light attenuation coefficient, was calculated according to Kirk [13] as: Kd = 1/z*ln (E_0_/E_z_) where E_0_ is the surface incident irradiance and E_z_ is the irradiance at depth z. Low Kd values describe transparent water with little attenuation of radiation, whereas high Kd values mean high suspended particles in the water column producing high radiation extinction.

### Sampling

At each site, subaquatic video profiles were taken perpendicular to the coast, from 15 m depth to the waterline by SCUBA diving. Video photography is a form of non-destructive sampling that does not require the removal of the organisms or interfere with the environment. The technique has also the advantage of allowing researchers to gather data quickly from remote or inhospitable places [14] as it is Antarctica in winter months. However, it can underestimate understory taxa when layering occurs. Each video was recorded using a digital camera Sony Mini DVD 108 (Carl Zeiss lens, optical zoom 40×, equipped with 0.5× wide angle conversion lens). Video transects were recorded 0.50 m above the bottom and constant artificial video light was used to decrease shade/overlaps and to identify small individuals. The length of transect belts was determined by the slope of the sea floor at each site. A snapshot of the video was captured every 30 seconds and coverage of macroalgae was estimated on the computer screen with an overlay of a 25 square grid, in which each square represented 4% of the total cover. Approximately 40 photographic samples were analyzed in each video transect. Photographic samples were clustered in ranges of depth: 0–2.99 m, 3–5.99 m, 6–8.99 m, 9–12.99 m, 13–15 m.

In the framework of the Research project PICTA N°7 (“Glacial retreat impact, due to global warming, on the benthic macroalgal distribution in Potter Cove”), the Environmental and Tourism Antarctic Management Program of the National Direction of the Antarctic (Dirección Nacional del Antártico) in the Republic of Argentina, has issued the appropriate permissions to all the stages of this research:

To the Specially Protected Area N°132 “Peninsula Potter” (under art. 7, Annex V of the Madrid Protocol, Law 25260)
Taking and harmful interference and introduction of species (under art. 3, Annex II of the Madrid Protocol, Law 24216)

Both of these permissions properly followed the regulations in force.

### Community Characterization

The newly ice-free areas were characterized by ecological indexes: diversity, richness and evenness were calculated for each range of depth. Diversity was determined by Shannon index: H = ∑ p_i_ log_2_ p_i_ where p_i_ is the relative abundance (estimated by cover data) of taxon i in the range of depth and evenness was calculated using Pileoús Index as J =  H/log_2_ S. Richness (S) was the total number of species. Abundance was estimated as percent cover data.

The macroalgal taxa were classified according to their life history (Table 1) in annuals, pseudoperennials (species living two years or re-growing during the second year) and perennials [15]. In Antarctica, the presence of perennial algae can be associated to more stable (and “mature”) communities that bring refuge and food to a rich community of invertebrates.

**Table 1:** Percentage cover of macroalgae species recorded in the study sites: mean (statistical error); absence in a given site is indicated by “−”.

Macroalgae species	S1	S2	S3	S4	S5	S6	Life history	Endemic	Cold adapted	Shade adapted
**Ulvophyceae**										
*Monostroma hariotii* Gain 1911	0.04 (0.04)	0.86 (0.54)	–	–	0.58 (0.29)	–	A			X
**Phaeophyceae**										
*Adenocystis utricularis* (Bory) Skottsberg 1907	0.10 (0.10)	0.08 (0.08)	0.23 (0.13)	–	–	–	A			
*Ascoseira mirabilis* Skottsberg 1907	6.77 (2.12)	4.23 (1.75)	1.50 (0.70)	0.34 (0.34)	–	–	P	X		
*Desmarestia anceps* Montagne 1942	12.64 (9.67)	10.00 (3.40)	4.37 (2.54)	1.72 (1.05)	–	0.83 (0.51)	P (s)	X	X	X
*Desmarestia antarctica* Moe & Silva 1989	0.95 (0.13)	3.89 (2.81)	–	2.15 (2.15)	–	–	A (s)	X	X	X
*Desmarestia menziesii* J. Agardh 1848	18.08 (8.02)	8.20 (3.14)	11.07 (6.78)	4.06 (3.56)	–	5.48 (3.12)	P (s)	X	X	X
*Himantothallus grandifolius* (Gepp & Gepp) Zinova 1959	3.03 (1.78)	10.86 (4.70)	19.79 (19.32)	14.01 (8.01)	–	6.44 (3.63)	P (s)	X	X	X
*Phaeurus antarcticus* Skottsberg 1907	0.56 (0.42)	0.11 (0.11)	0.11 (0.11)	0.74 (0.21)	–	0.20 (0.20)	A (s)	X	X	X
**Rhodophyceae**										
*Ballia callitricha* (C. Agardh) Kützing 1843	0.28 (0.18)	–	0.29 (0.29)	–	–	–				
*Corallinaceae (2 genera Lithothamnion- Hydrolithon)*	7.90 (0.76)	3.09 (1.51)	5.05 (2.83)	2.04 (1.87)	–	4.60 (3.03)				
*Curdiea racovitzae* Hariot 1900	–	0.15 (0.15)	–	–	–	–		X		X
*Georgiella confluens (*Reinsch) Kylin 1956	0.51 (0.51)	–	–	–	–	–		X	X	X
*Gigartina skottsbergii* Setchell & Gardner 1936	13.72 (2.10)	7.83 (2.55)	9.33 (5.29)	11.84 (8.56)	–	2.00 (1.22)	PsP		X	X
*Hymenocladiopsis crustigena* Moe 1986	–	–	–	–	–	0.08 (0.08)			X	X
*Iridaea cordata* (Turner) Bory 1826	7.76 (1.04)	5.49 (1.69)	6.81 (3.68)	11.72 (5.12)	–	5.42 (3.46)	PsP		X	X
*Myriogramme spp or Neuroglossum ligulatum*(Reinsch) Skottsberg in Kylin and Skottsberg1919	–	0.15 (0.09)	0.47 (0.28)	–	–	–	PsP			
*Palmaria decipiens* (Reinsch) Ricker 1987	0.29 (0.29)	–	0.04 (0.04)	–	24.43 (8.85)	0.37 (0.24)	PsP		X	
*Plocamium cartilagineum* (Linné) Dixon 1967	1.27 (1.70)	2.90 (1.12)	4.97 (2.29)	2.89 (2.34)	–	4.61 (2.18)			X	X
*Trematocarpus antarcticu*s (Hariot) Fredericq &R.L.Moe 2009	–	–	–	–	–	1.21 (1.21)	p	X		X
Total Cover	73.74 (6.46)	57.85 (6.38)	64.02 (10.38)	51.52 (14.85)	25.01 (9.06)	31.81 (7.49)				

Macroalgae are classified according to their life history in annual (A), pseudoperennial (PsP) and perennial (P) species [15], (s) indicates sporophyte phase. Shade, cold adapted and endemic species are marked with a cross.

One way ANOVA were performed to test the effects of the site on the analyzed variables. Post-hoc multiple means comparisons were analyzed using Tukey Test. Homogeneity of variances was checked using Cochran’s Test. Tests were performed with Statistica TM 6.0 software package.

## Results

**Figure 2:**
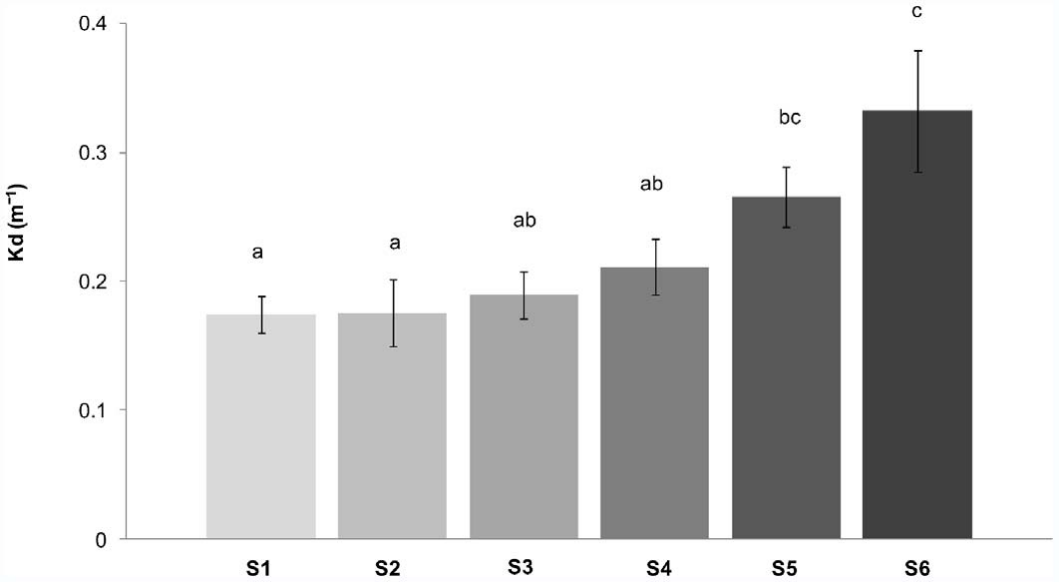
Light attenuation coefficient (Kd) calculated for the studied sites (S1: site 1, S2: site 2, S3: site 3, S4: site 4, S5: site 5 and S6: site 6) in summer (December 2009 to March 2010). Kd = 1/z*ln (E_0_/E_z_) where E_0_ is the surface incident irradiance at 0 m and E_20_ is the irradiance at 20 m depth. Photosynthetic active radiation (PAR) was measured weekly at noon (units: µmol m^−2^ s^−1^).

In summer, significant differences of Kd were found between the studied sites (Figure 2). Kd for S6 was significantly higher compared to S1, S2, S3 and S4, while Kd for S5 was significantly higher compared to S1 and S2 (1-way ANOVA, p<0.05, Tukey test).

Macroalgae colonized all the studied sites. A total of 18 species of macroalgae and two genera of encrusting red algae (Corallinaceae) were identified (Table 1).

**Figure 3:**
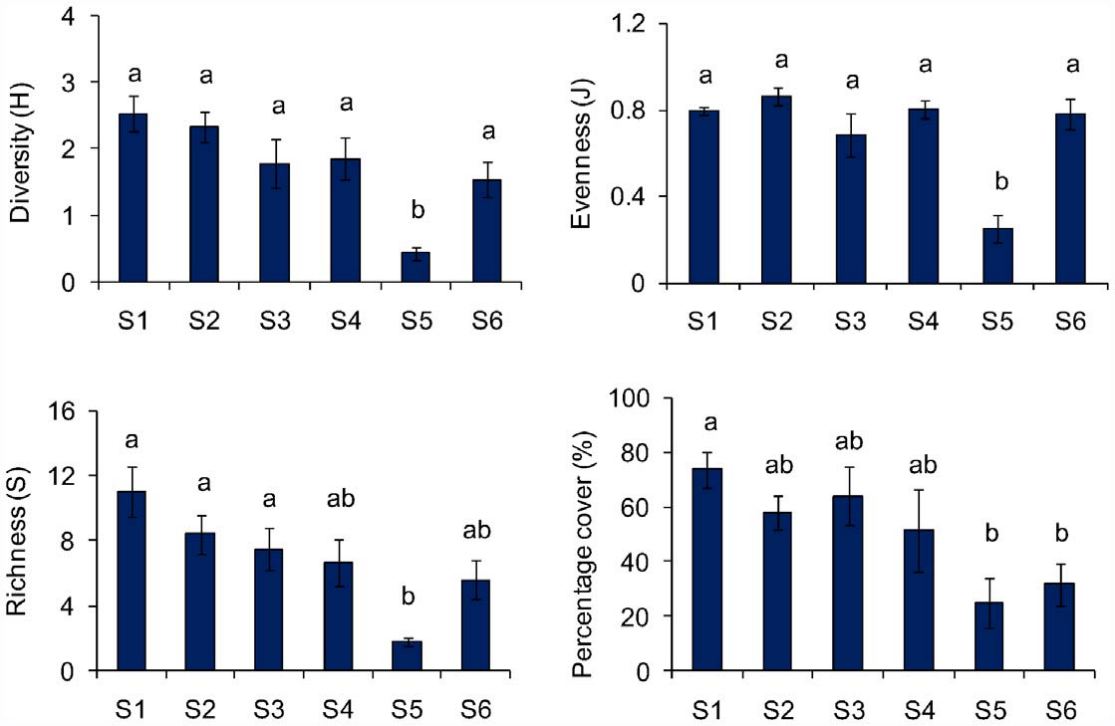
Macroalgal diversity, richness, evenness and total cover at the six study site: (S1: site 1, S2: site 2, S3: site 3, S4: site 4, S5: site 5 and S6: site 6. Lowercase letters indicate significant differences between the sites, i.e. **a** is significant different from **b.**

Diversity and evenness were lower in S5 compared to the rest of the studied sites (1-way ANOVA, p<0.05, Tukey test, Figure 3). In addition, richness was significantly lower in S5 compared to S1, S2 and S3.

Total macroalgal cover was significantly different among sites (1-way ANOVA, p<0.05, Figure 3). The highest cover was found in S1, whereas the lowest was recorded for S5 and S6 (Tukey Test, p<0.05, Table 1, Figure 3).

**Figure 4:**
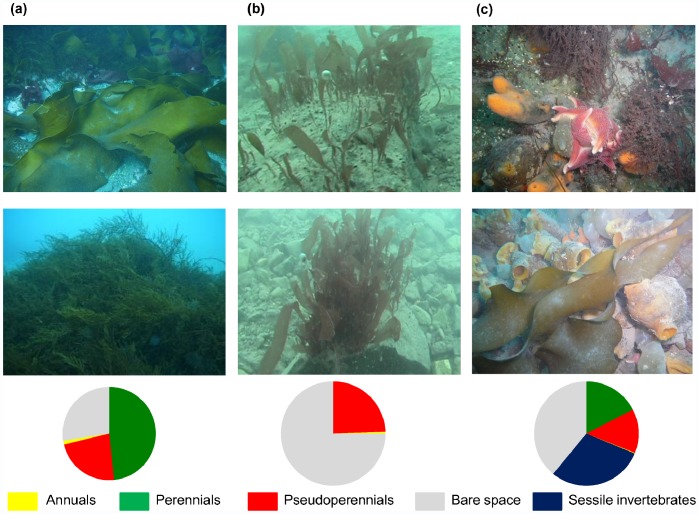
Three different situations (a) Site1, with mature macroalgal communities (b) Site 5, were the red algae species *Palmaria decipiens* was dominant, and recorded under high sediment load conditions and (c) Site 6 (island), with coexistence of macroalgae and macrofauna. Taxa percentage cover (%) at the three sites are shown; macroalgae were classified according to their life history in annuals, pseudoperennials and perennials (Figure 1).

S1 to S3 were dominated by perennial macroalgae, mainly large brown of the Order Desmarestiales (*Desmarestia anceps, D. menziesii* and *Himantothallus grandifolius* (Table 1, Figure 4). In S4 and S6 there was an almost equal contribution of perennial and pseudoperennial macroalgae (Table 1, Figure 4). In S6 there was also a sessile macrofaunal community (Figure 4). Finally, S5 was dominated by the pseudoperennial *Palmaria decipiens*, with a lower presence of *Monostroma hariotii*, an annual green algal species (Table 1, Figure 4).

## Discussion

Macroalgae are colonizing the new available substrate of Potter Cove showing an expansion of the distribution observed 17 years ago, when they were located between the rocky mouth (entrance) and S1 [9].

The 20 taxa recorded in this study constitute a low number compared to the registered macroflora of Potter Cove, which includes 42 species [9]. This low richness can be attributed to several causes like the recent development (15 years approximately) of the newly ice-free areas, the level of stress and disturbance-due to the reduction of the light penetration and ice disturbance, and site-specific available substrate.

### Observed Patterns and Possible Explanations

#### I. The complexity of the macroalgal community is positively correlated to the elapsed time from the ice retreat

S1, S2 and S3 were the first to be available for benthic colonization and it is plausible that macroalgal communities have been developing there for a longer time period. Hence, a higher macroalgal cover and more mature communities are found there. Indeed, a high contribution of large brown species of order Desmarestiales (approximately 30% of the macroalgal cover) was registered in these three sites. This macroalgal composition was similar to the sites situated on the outer Cove [9], [10].

On the other hand, S5 and S6 were ice-released later in time and a less mature macroalgal community would be expected there.

However, an older age of the site is not always related to the maturity of the community. Indeed, S5 is older than S6 and shows a significantly lower diversity. Other factors should also be considered to explain the observed patterns (see below).

#### II. Macroalgal development depends on the optical conditions and the sediment input in the water column

Light availability is a major factor determining the vertical distribution limits of subtidal macroalgae in Antarctica [16]. There was a positive relation between light penetration and macroalgal cover. Higher light transmittance was related to a more complex community (in terms of a higher contribution of large perennial macroalgae) and a higher cover (S1), whereas less mature communities and a lower cover occurred in the sites with the highest sediment input (S5 and S6) [17].

In addition, glacial run-off not only reduces light penetration but also causes accumulation of sediment on the sea floor which may limit the attachment of benthic algae [18], [19], [20]. In the Baltic Sea, sedimentation is an important factor affecting the colonization and development of the macroalgal community, as sediment removal increased the total cover of the macroalgal vegetation [21].

At S5, the video images showed *Palmaria decipiens* as by far, the dominant species, immersed in a high sediment load. This species seemed to be able to cope with both low light penetration and high sediment accumulation, probably due to its physiological plasticity [22], [23]. Moreover, this site was the only one where encrusting red coralline algae were not recorded. The high input of sediment forms a dense carpet on the seafloor, either preventing the development of this group or impeding its detection, if present.

Even though S5 and S6 had similar optical conditions, S5 showed significantly lower diversity than S6. Thus, other factors should also be considered, as ice disturbance (see below) to better explain the observed patterns.

#### III. Macroalgal colonization is negatively affected by the ice disturbance

Ice scouring can be a strong driver of the distribution and abundance of macroalgae, affecting zonation and diversity patterns in Polar Regions [24]. Although there are no studies assessing the ice effect in the study sites, personal observations identified that S5 and S6, which are the sites with the lowest percent cover of macroalgae, are probably the most ice-disturbed areas. Among these, S5 could be the most disturbed area as it is closer to the glacier, where landslides of ice blocks impact directly on the sea floor (pers. observation). Thus, on S6 the higher diversity of the community could be attributed to a lower ice-scouring disturbance. Nevertheless, the differences in substrate and slope may also contribute to explain the patterns found in S5 and S6.

#### IV. Macroalgal colonization is determined by substrate and slope

Substrate and slope play a key role in determining algal settlement [10]. The studied newly ice-free areas have shown to provide suitable hard substrate which is essential for the attachment of macroalgae and macroalgal cover was observed in the inner side of the Cove, where it was historically devoid of them [9].

Substrate and slope could also explain the differences between S5 and S6.

These sites undergo high sediment input conditions but the vertical wall of the island prevents sediment accumulation. Vertical substrate was shown to favor the establishment of sessile macrofaunal communities [25]. This pattern was also found on S6, resulting in the coexistence of macroalgae and macrofauna. At shallow depths, macroalgae are dominant, at intermediate depths (between 6 and 12 m) there is an even and relevant coexistence between macroalgae and macrofauna, and from 12 m depth and deeper, invertebrates dominate the cover of the rocky slopes (unpublished data). Furthermore, the high decrease of macroalgal abundance beyond 12 m depth could be determined by the low light availability for photosynthesis under 10 m depth [23]. The high diversity of invertebrates (filter feeders, herbivorous and scavengers) seemed to be favored by the hard substrate, vertical wall arrangement [26] and due to the strategic location of the island in the Cove: speed and direction of the water current movements and the resuspension of the organic particles could be providing propitious habitat for their development with time [25].

A special consideration is necessary for the encrusting red algae (Corallinaceae). In many quantitative studies of subtidal communities, this group is mentioned as “encrusting coralline algae” on hard substrate, without a complete taxonomic species identification. Some authors emphasize the importance of their presence, primarily because they will provide future answers of the evolution of the acidification of the ocean [27], [28]. Barnes *et al.* [29] considered that coralline algal presence is a useful surrogate measure of the relative change in turnover or disturbance rate with substrate surface area and depth. Other studies showed shade adaption [30], high sensitivity to canopy loss [31] and absence of this group in sites exposed to sedimentation [19]. The presence of Corallinaceae in most of the studied newly ice-free areas (they were recorded in all sites except for S5) could be related to their adaptation to low light requirements. However, the cover of this group could have been underestimated due to the limitation of the methodological approach, as a high substrate occupation was recorded in other Antarctic sites [32]. Their cover quantification in Potter Cove, including the quantification of the individuals beneath the canopy, will provide valuable information as a starting point for future research and monitoring.

**Figure 5:**
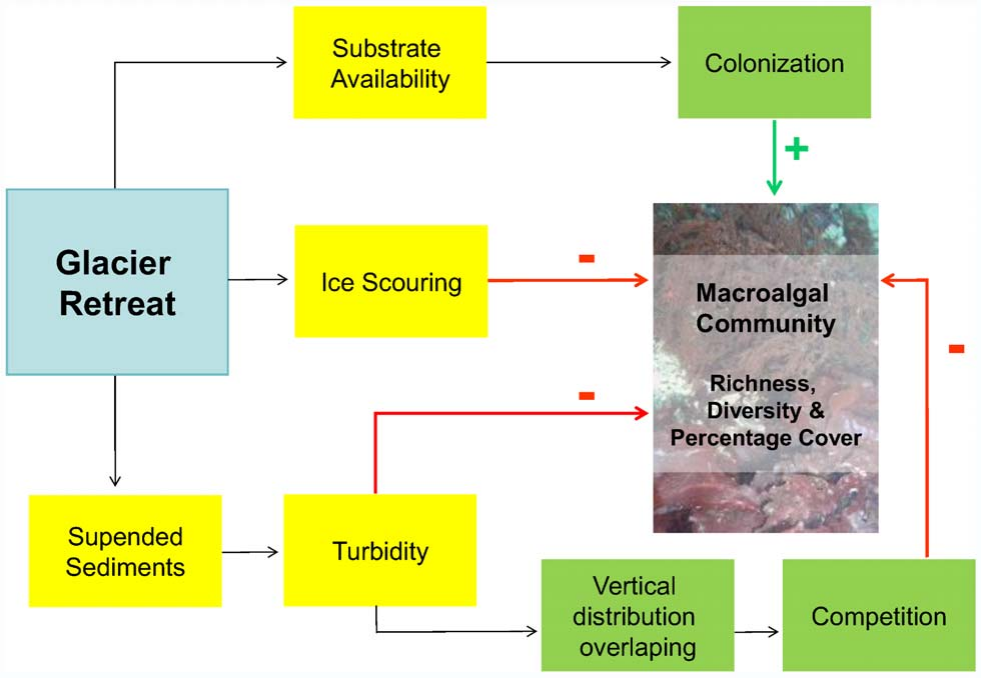
Conceptual model for newly ice-free areas of Potter Cove.

To explain the pattern of spatial distribution of macroalgal species, a conceptual model was developed (Figure 5). The ice melting is the primary cause of changes in the macroalgal communities in the inner Cove, and it could be mediated by different associated phenomena. Ice retreat originates newly ice-free areas providing substrate availability for benthic colonization. The substrate availability is positively related to diversity, richness and macroalgal cover. In addition, glacier melting increases ice scouring or impact, having a negative effect on these parameters. Finally, melting increases the amount of sediments in the water column and enhances turbidity. This effect has a direct negative impact on the mentioned parameters because algae have not sufficient light availability for photosynthesis. Moreover, there might be an indirect effect caused by sediments: macroalgae may shift their vertical distribution increasing overlapping and competition, resulting in a negative effect.

Macroalgae are an important food source for fish and benthic invertebrates in Potter Cove [33], [34]. Furthermore, as Potter Cove is usually a very low phytoplankton biomass accumulation system [3], macroalgal biomass production of the outer cove was proposed to be the main carbon source for the rich benthic fauna present in the area [6]. Tatián *et al.* [11] found macroalgal debris in the gut contents of ascidians and other suspension feeders of the benthic community. Considering that the spatial distribution of the macroalgal community is expanding to the inner side of the Cove, the main question is how this new available biomass would contribute to the coastal food web.

### Future Perspectives

Smale and Barnes [4] emphasized that following glacier retreat, benthic colonization and succession processes commence. However, it is currently unclear to what extent. Succession field experiments are being carried out to follow the development of the communities in natural habitats.
